# Characterization of Nanoreinforcement Dispersion in Inorganic Nanocomposites: A Review

**DOI:** 10.3390/ma7064148

**Published:** 2014-05-28

**Authors:** Nouari Saheb, Najam Ul Qadir, Muhammad Usama Siddiqui, Abul Fazl Muhammad Arif, Syed Sohail Akhtar, Nasser Al-Aqeeli

**Affiliations:** Department of Mechanical Engineering, Center of Research Excellence in Nanotechnology, King Fahd University of Petroleum and Minerals, Dhahran 31261, Saudi Arabia; E-Mails: g201204240@kfupm.edu.sa (N.U.Q.); g200904210@kfupm.edu.sa (M.U.S.); afmarif@kfupm.edu.sa (A.F.M.A.); ssakhtar@kfupm.edu.sa (S.S.A.); naqeeli@kfupm.edu.sa (N.A.-A.)

**Keywords:** nanoreinforcement, dispersion, matrix, qualitative characterization, quantitative characterization, nanocomposites, nanomaterials

## Abstract

Metal and ceramic matrix composites have been developed to enhance the stiffness and strength of metals and alloys, and improve the toughness of monolithic ceramics, respectively. It is possible to further improve their properties by using nanoreinforcement, which led to the development of metal and ceramic matrix nanocomposites, in which case, the dimension of the reinforcement is on the order of nanometer, typically less than 100 nm. However, in many cases, the properties measured experimentally remain far from those estimated theoretically. This is mainly due to the fact that the properties of nanocomposites depend not only on the properties of the individual constituents, *i.e.*, the matrix and reinforcement as well as the interface between them, but also on the extent of nanoreinforcement dispersion. Therefore, obtaining a uniform dispersion of the nanoreinforcement in the matrix remains a key issue in the development of nanocomposites with the desired properties. The issue of nanoreinforcement dispersion was not fully addressed in review papers dedicated to processing, characterization, and properties of inorganic nanocomposites. In addition, characterization of nanoparticles dispersion, reported in literature, remains largely qualitative. The objective of this review is to provide a comprehensive description of characterization techniques used to evaluate the extent of nanoreinforcement dispersion in inorganic nanocomposites and critically review published work. Moreover, methodologies and techniques used to characterize reinforcement dispersion in conventional composites, which may be used for quantitative characterization of nanoreinforcement dispersion in nanocomposites, is also presented.

## 1. Introduction

Composite materials are made of two or more different phases, *i.e.*, matrix and reinforcement(s) with a clear interface between them. Inorganic composites are those composites where the matrix is a metallic or ceramic phase. Metal matrix composites (MMCs) combine the good ductility and toughness of the metal matrix and the high strength and stiffness of the ceramic reinforcement [1], this made MMCs candidate materials in many automotive and aerospace applications. Monolithic ceramics have high mechanical strength, superior temperature stability and good chemical durability but exhibit low fracture toughness because of their ionic or covalent bonding which limited their use in many industrial sectors. This was the driving force to develop ceramic matrix composites (CMCs) where several approaches such as transformation toughening, ductile-phase toughening and reinforcement toughening have been adopted to improve the fracture toughness of ceramics [2].

**Figure 1:**
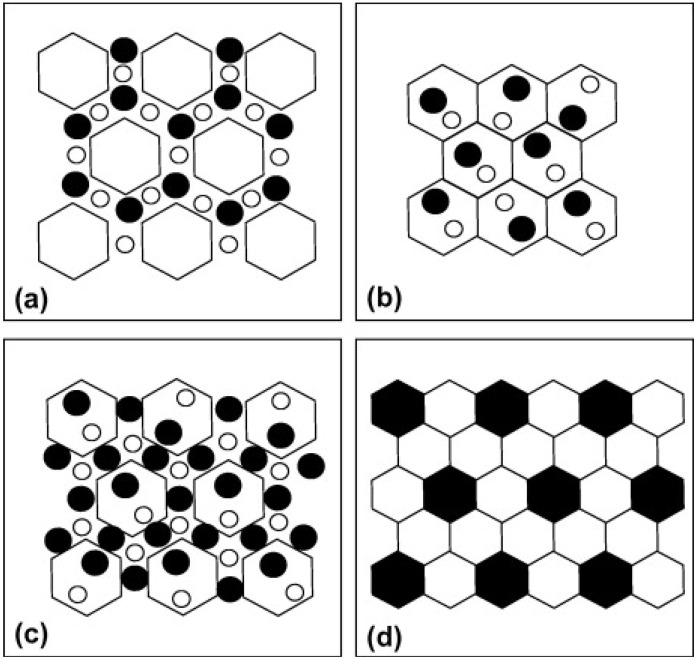
Possible distribution of the matrix and reinforcement phases in a nanocomposite. The reinforcement phase is distributed (**a**) along the grain boundaries of the matrix; (**b**) inside the grains of the matrix; (**c**) both inside the grains and along the grain boundaries; or (**d**) both the matrix and reinforcement grains are uniformly distributed [1].

Despite the fact that particle reinforced MMCs exhibit isotropic properties and have high strength and stiffness, their ductility and toughness are reduced because of the large size of particles which leads to easy initiation and propagation of cracks in the ceramic particles or at the interface. Fortunately, reducing the size of the reinforcement to less than about 100 nm, in metal matrix nanocomposites (MMNCs), brought about significant improvement in ductility and toughness with simultaneous increase in strength [1,3]. On the other hand, decreasing the grain size of ceramics to the nanometer scale was reported to increase their hardness and fracture strength and decrease their fracture toughness [1,4]. However, the improvement of fracture toughness was possible through the addition of a reinforcing phase to the ceramic matrix as in ceramic matrix nanocomposites (CMNCs) where the reinforcement has nanoscale dimensions. Suryanarayana and Al-Aqeeli [1] classified the possible distribution of the reinforcement and matrix phases in a nanocomposite as shown in Figure 1. The reinforcement phase is distributed along the grain boundaries of the matrix (a); inside the grains of the matrix (b); both inside the grains and along the grain boundaries (c); or both the matrix and reinforcement grains are uniformly distributed (d).

The two most noticeable advantages of MMNCs and CMNCs are (a) the extraordinarily high strength to weight ratio that is attainable in these nanocomposites compared to MMCs and CMCs; and (b) the relatively much greater matrix-to-reinforcement load transfer efficiency that is possible due to the extremely higher surface area to volume ratio of nanoreinforcements used in nanocomposites compared to macro-scale reinforcements traditionally employed in MMCs and CMCs. However, having said this, both types of nanocomposites have been repeatedly reported to pose difficulties during fabrication process owing to the inherent tendency of nano-size reinforcements to agglomerate during solid-state as well as liquid-state processing [5,6,7]. Since the bulk mechanical properties of a composite depend on the extent of dispersion of the embedded reinforcement, agglomeration of nanoreinforcement is certain to lead to property fluctuation across the cross-section of the tested sample, the degree of fluctuation being directly dependent on the degree of agglomeration. Apart from introducing necessary modifications in the fabrication process in order to minimize the occurrence of nanoreinforcement agglomeration, equally significant are the types of characterization techniques used to study the extent of dispersion of the nanoreinforcement phase in the nanocomposite. The techniques that have been utilized so far, for analyzing both the nature and quality of nanoreinforcement distribution, have been reported to only present the distribution in a certain localized region of the entire cross section of the nanocomposite sample [5,8,9,10,11,12,13,14]. Moreover, a wide majority of these characterization techniques have been used to assess the nanoreinforcement distribution on a qualitative basis only, providing no means to quantify the extent of distribution, which is highly desirable for a more effective and reliable prediction of bulk mechanical properties of the nanocomposite. Although excellent review papers were published on the processing, characterization, and properties of polymer matrix nanocomposites [15,16,17,18,19,20,21,22,23], less literature is available on metal and ceramic matrix nanocomposites [1,2,9,24,25,26,27,28,29,30]. On top of that, the issue of nanoreinforcement dispersion in metal and ceramic nanocomposites was not fully addressed. The objective of this review is to provide a comprehensive description of qualitative and quantitative characterization techniques used to evaluate the extent of nanoreinforcement dispersion in metal and ceramic matrix nanocomposites and critically review published work in this area.

## 2. Qualitative Characterization

### 2.1. Characterization Techniques

Techniques used for qualitative characterization of nanoreinforcement distribution in nanocomposites can be classified into two broad categories—(a) techniques which directly yield a two-dimensional or three-dimensional image showing the nanoreinforcement and the matrix phases in the same way they exist in the real nanocomposite (direct analysis techniques); and (b) techniques which measure a specific physical property of either or both of the phases of the nanocomposite, with the nanoreinforcement distribution being predicted indirectly from the results of the measurement (indirect analysis techniques). Moreover, some of these techniques can only be used when the nanocomposite exists in a bulk or consolidated form, while others can be applied to yield distribution information regardless of whether the nanocomposite exists in the form a powder or in a consolidated state.

Scanning Electron Microscopy (SEM) and Transmission Electron Microscopy (TEM) have been frequently used for qualitative analysis of nanoreinforcement distribution in polymer matrix [31,32,33], ceramic matrix [8,9,11], and metal matrix nanocomposites [12,34,35]. Despite offering dual advantages of obtaining very high resolution topographic images and compositional maps of the elements present in the matrix, both techniques suffer from a common drawback of analyzing a highly localized portion compared to the entire bulk volume of the nanocomposite. This common disadvantage of SEM and TEM is further superimposed by the inherent deficiency of either of these techniques to obtain a three-dimensional image of the nanocomposite sample. A detailed description concerning the architecture and operating principles of SEM and TEM techniques can be found in [36,37] respectively.

X-ray mapping or compositional imaging of the distribution of elements is one of the major capabilities of electron beam microanalysis since it frees the operator from the necessity of making decisions about which image features contain elements of interest [38]. X-ray mapping in SEM and Electron Probe Micro-Analysis (EPMA) may be applied to bulk specimens at a spatial resolution of about 1 µm. X-ray mapping of thin specimens in TEM or Scanning Transmission Electron Microscopy (STEM) may be accomplished at a spatial resolution ranging from 2 to 100 nm, depending on specimen thickness and the microscopic resolution [38]. X-ray maps are formed by collecting characteristic X-rays emitted from elements in the specimen when a primary electron beam is incident on its surface. Electrons are ideal for generating X-ray compositional maps because they can be focused to a small probe, they can be deflected to form a scanned beam raster, and they can excite atoms in the sample to produce characteristic X-ray signals [38]. X-ray mapping has therefore been used with exactly the same applicability towards ceramic matrix [7,39] and metal matrix [13,14] nanocomposite systems, to generate composition maps of the various constitutive elements present in the matrix. More information on the technique can be found in [40].

Detailed description of Atomic Force Microscopy (AFM) was provided by Eaton [41]. The technique is capable of providing a three-dimensional profile of a surface on a nano scale by measuring forces between a sharp probe (less than 10 nm diameter), and the surface at a very short distance (0.2–10 nm probe-surface separation). The major difference in various types of AFM architectures lies in the different ways in which the forces between the probe and the sample surface are monitored. Since AFM image is generated from the interaction forces between the probe and the sample surface, its resolution strongly depends on the accuracy with which these forces are measured. Hence, if the nanocomposite sample consists of nanoreinforcements, which are smaller than the probe diameter, details of such reinforcements are highly probable to be omitted in the AFM image. Despite facing this architectural deficiency, AFM has still been successfully used to provide a qualitative analysis of the nanoreinforcement distribution in polymer matrix [31,32,42], metal matrix [43,44], and ceramic matrix [5,12] nanocomposites.

Deing *et al.* [45] outlined the architecture, operational principles, and application of Confocal Raman microscopy technique, which has been proven to be an extremely popular analytical technique for qualitative analysis of nanoreinforcement distribution in nanocomposites. It offers unique advantages such as lack of sample preparation, very high image resolution, outstanding composition contrast of various elements present in the matrix, and ability to perform three-dimensional mapping of bulk nanocomposite samples. The technique has been used for nanoreinforcement dispersion analysis in polymer matrix [46,47], and ceramic matrix nanocomposites [7,48].

Small angle scattering techniques [40], such as small angle X-ray scattering (SAXS) and small angle neutron scattering (SANS), are capable to give information on the structural features of particles of colloidal size, as well as their spatial correlation. Both SAXS and SANS are powerful techniques for determining size, shape, and internal structure of particles in the size range from few nanometers up to about hundred nanometers. SAXS is an elastic scattering of X-rays from the electrons in atoms and therefore it is sensitive to electron density fluctuations in the sample, due to which it offers the advantage of an extremely high resolution capable of detecting atomic size features. This made SAXS a highly attractive tool for analyzing spatial distribution of nanoreinforcements, of the order of only a few nanometers, in nanocomposites. It has been most widely used for qualitative analysis of nanoreinforcement distribution in polymer matrix nanocomposites [49,50,51], and ceramic matrix nanocomposites [52,53].

Particles dispersed in a continuous electrolyte solution are in constant Brownian motion. When two particles approach each other in an electrolytic suspension, the energy between the particles determines whether the particles will agglomerate or remain as distinct entities in the suspension. Generally particle agglomeration occurs when larger attractive than repulsive forces exist between them. At higher ionic strength of the electrolyte or lower density of surface charges induced on particle surfaces, the particles may agglomerate irreversibly. However, if the density of similar surface charges on the particle surfaces can be increased by altering the pH of the electrolyte, net repulsive forces can be introduced between the particles, resulting in a higher density of dispersed than agglomerated particles. The zeta potential [54], which is a quantitative measure of the particle surface charge, gives an indication of the stability of a colloidal system. A higher absolute value of zeta potential induces a lower degree of particle agglomeration, leading to a higher concentration of non-aggregated particles in the electrolyte. Since zeta potential of the dispersed particles is a function of the pH value of the electrolyte, the degree of particle agglomeration also depends strongly on the current pH value. Hence an optimum value of pH can be found which corresponds to a highest absolute value of zeta potential and consequently maximum dispersion of the particles in the electrolyte. With reference to nanoreinforcement distribution in nanocomposites, zeta potential measurement offers a two-fold advantage—(a) it can be used to find the value of electrolyte pH at which the nanoreinforcement particles exhibit maximum dispersion, and the same value of the pH is then used for the actual fabrication process of the nanocomposite; and (b) if the surface of the nanoreinforcement particles is functionalized with negatively charged functional groups, an optimum value of pH can be found at which they not only experience maximum repulsion amongst themselves, but also bear maximum electrostatic attraction with the positively charged co-existing metal ions, due to dissociation of the matrix precursor, also suspended in the same electrolyte. Hence, it can be concluded that zeta potential measurement technique offers the advantage of facilitating maximum nanoreinforcement-matrix bond-strength, apart from maximizing the homogeneous nanoreinforcement distribution in the nanocomposite. As a result, the technique has been successfully used as a pre-fabrication characterization technique in polymer matrix [55,56,57,58], metal matrix [6], and ceramic matrix nanocomposite systems [9,11,59,60].

With the recent developments of new three-dimensional (3-D) characterization tools, a clear and accurate qualitative analysis of nanoreinforcement distribution in nanocomposites became possible. However, the majority of techniques which have been used for visualization of 3-D microstructural images rely on serial-sectioning of the sample which is both destructive and time-consuming. X-ray microtomography (microCT) is an excellent technique that eliminates destructive cross-sectioning, and allows for superior resolution and image quality demanding minimal sample preparation. MicroCT is a 3-D radiographic imaging technique capable of achieving a spatial resolution close to 1 µm unlike conventional CT tomography systems, which offer a maximum achievable resolution of 1 mm. In both conventional tomography and microtomography, hundreds of 2-D projection radiographs of the specimen are taken at many different angles. The information contained in a radiograph is a projection of the absorption density inside the sample onto a plane perpendicular to the direction of the X-ray beam. If the sample is imaged several times in different orientations, a 3-D bulk information on the sample structure can be obtained using computer algorithms [61]. With the most recent introduction of X-ray nanotomography (nanoCT) systems [62,63], 3-D image construction of nanostructured materials will now also be possible. However, evidence of a real-time application of nanoCT systems in qualitative analysis of functionally graded nanostructured materials has yet to be reported. Hence the inclusion of X-ray tomography as a characterization technique in this review, for qualitative analysis nanoreinforcement distribution in nanocomposites, is solely based on its inherent potential to resolve the nanoreinforcement phase, not because it has already been used for this purpose. Use of microCT for qualitative analysis was reported for polymer matrix [64,65], metal matrix [66,67], and ceramic matrix composite systems [68]. A detailed description concerning the underlying operational principles and architecture of this technique can be found in [69].

Table 1 summarizes most important characterization techniques used for qualitative analysis of nanoreinforcement distribution in inorganic nanocomposites. The mode of analysis for each technique, physical state of the nanocomposite, and extent of nanoreinforcement distribution are given. In addition, it includes information about whether each technique can only be used to obtain two-dimensional distribution information in a localized area of nanocomposite, or an overall three-dimensional picture of the distribution in the bulk nanocomposite. All characterization techniques listed in Table 1 only yield distribution information when applied to the prepared nanocomposite, with the exception of zeta potential measurement technique, which only results in distribution information when applied individually to the nanoreinforcement and the matrix phases.

### 2.2. Metal Matrix Nanocomposites

**Figure 2:**
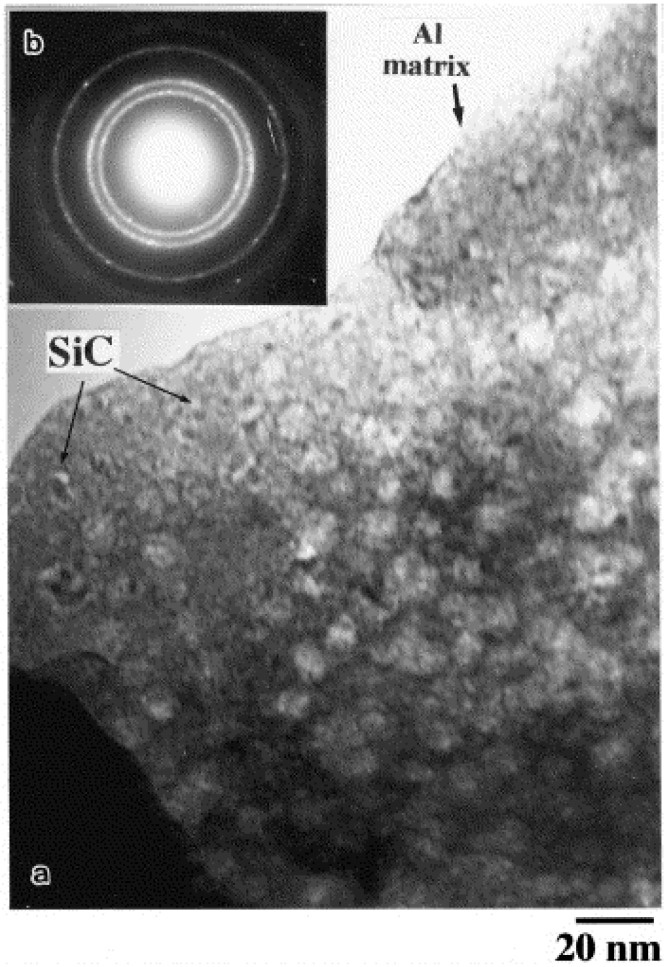
(**a**) BFI and (**b**) the corresponding SADP of mechanically solid state mixed SiC10/Al90 composite particle after 86 ks of ball-milling time [35].

**Figure 3:**
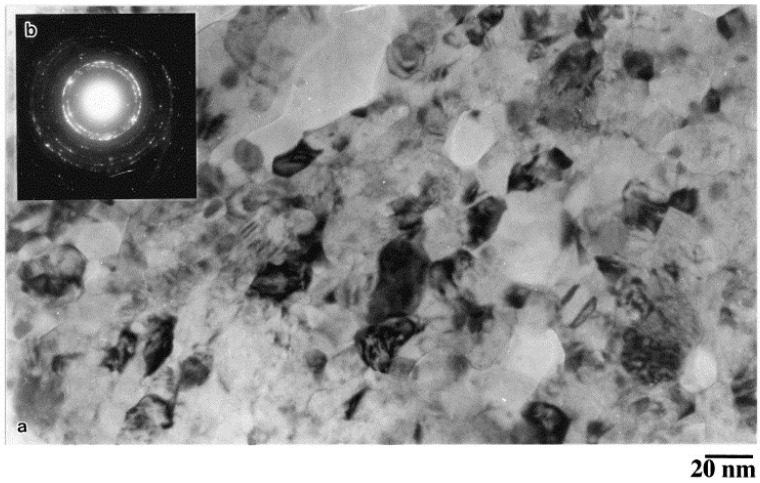
(**a**) BFI and (**b**) the corresponding SADP of as-consolidated mechanically solid state mixed SiC10/Al90 that were ball-milled for 86 ks [35].

El-Eskandarany [35] prepared SiC particle reinforced aluminum matrix nanocomposites, with varying volume fraction of SiC particles, using high-energy ball milling for homogeneous mixing of the SiC and Al powders, followed by consolidation of the nanocomposite powders using plasma activated sintering process. Figure 2 shows TEM micrograph of the Al-SiC nanocomposite powders containing 10 vol% SiC particles. As evident, the use of high-energy ball milling led to a highly homogeneous dispersion of SiC particles in the nanocomposite powders. Figure 3 shows TEM micrographs of the as-consolidated nanocomposite containing 10 vol% SiC particles, and also depicts a very well-dispersed SiC particles within the alumnium matrix.

**Table 1:** Qualitative characterization techniques.

Characterization technique	Mode of analysis	Sample form	Extent of distribution
Scanning Electron Microscopy (SEM)	direct	powder and bulk	localized (2D)
Transmission Electron Microscopy (TEM)	direct	powder and bulk	localized (2D)
Atomic Force Microscopy (AFM)	direct	bulk	localized (3D)
X-ray Microcomputed Tomography	direct	bulk	localized (3D)
X-ray mapping	direct	powder and bulk	localized and bulk
Zeta Potential Measurements	indirect	powder	localized and bulk
Raman Confocal Microscopy	direct	bulk	localized and bulk (3D)
Ultra-Small Angle X-ray Scattering (USAXS)	indirect	powder and bulk	–

**Figure 4:**
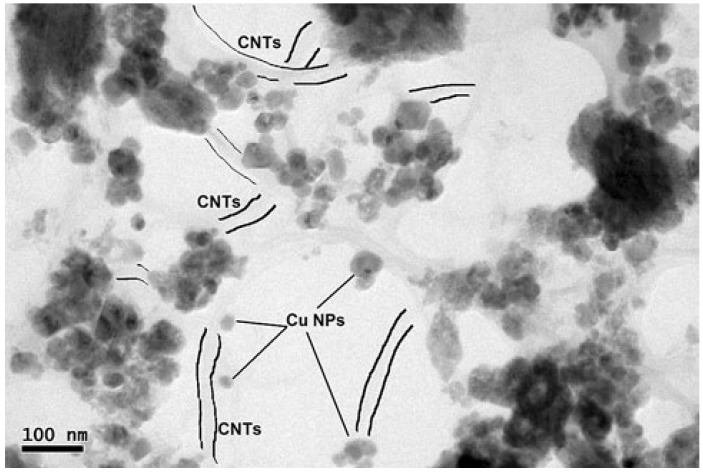
HRTEM micrograph of as-consolidated CNT reinforced Cu matrix nanocomposite fabricated via molecular level mixing and vacuum sintering technologies [34].

Lal *et al.* [34] fabricated Carbon nanotube (CNT) reinforced Cu matrix nanocomposites using molecular level mixing (MLM) of functionalized CNTs with aqueous CuSO_4_ solution, followed by high-energy ball milling (BM) of dried nanocomposite powders, which were then cold compacted and vacuum sintered for consolidation into the final nanocomposite. The nanocomposite powders prepared using a combination of MLM and BM were compared with those prepared using either only BM or only MLM, and the comparison revealed a highly dispersed morphology of CNTs in the nanocomposite powders prepared using both MLM and BM relative to the dispersion obtained using only BM. Figure 4 shows a TEM micrograph of the as-consolidated nanocomposite showing a highly homogeneous dispersion of CNTs in the Cu matrix by virtue of the combination of MLM and BM processes used in the fabrication of nanocomposite powders.

Park *et al.* [70] fabricated Al_2_O_3_ particle reinforced Fe matrix nanocomposite using a high frequency induction heated sintering method, starting from mechanically activated powders of Fe_2_O_3_ and FeAl. The authors examined X-ray mapping images of the as-consolidated nanocomposite and found that Al_2_O_3_ particles were well-dispersed inside the Fe matrix.

**Figure 5:**
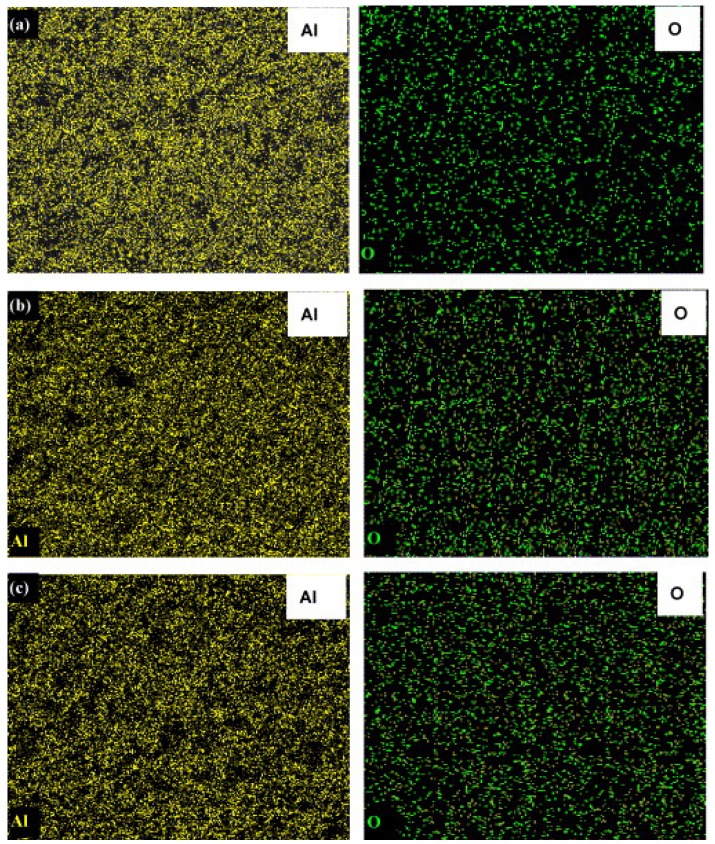
X-ray maps showing Al (left) and O (right) for the Al–Al_2_O_3_ (50 nm) powders milled for 20 h: (**a**) 20 vol% Al_2_O_3_; (**b**) 30 vol% Al_2_O_3_; and (**c**) 50 vol% Al_2_O_3_ [13].

Prabhu and co-workers [13] prepared homogenous Al-Al_2_O_3_ nanocomposite powders by high-energy milling. They used three different Al_2_O_3_ powder particle sizes, *i.e.*, 50 nm, 150 nm and 5 μm at volume fraction ranging between 20% and 50%. X-ray mapping of the mechanically milled powders confirmed uniform distribution of the reinforcement phase. The mapping spectra for the elements aluminum and oxygen in the Al–Al_2_O_3_ (50 nm) system for the three different volume fractions are presented in Figure 5. The authors reported similar results for 150 nm and 5 μm particle sizes milled under same conditions with different volume fractions.

**Figure 6:**
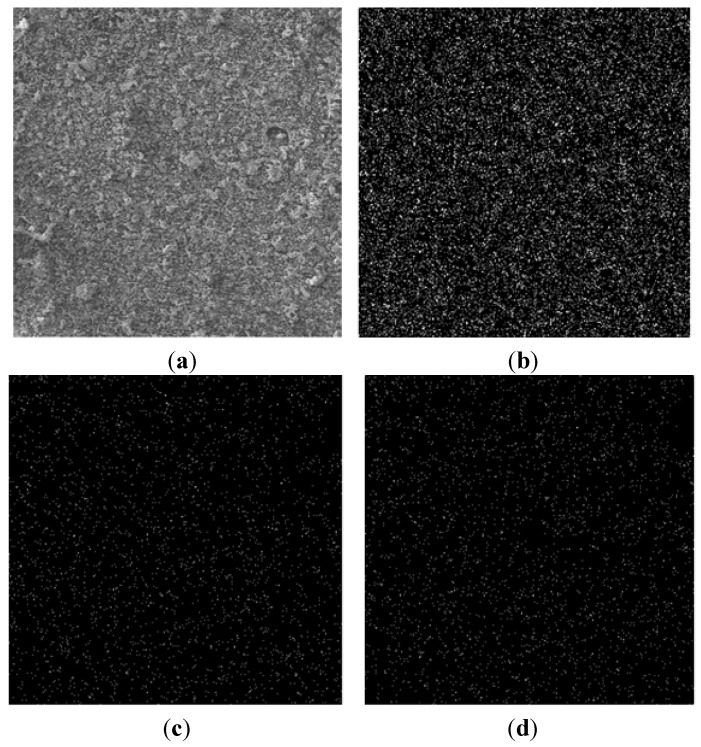
X-ray mapping of Ni-10wt%Al_2_O_3_ nanocomposite powder ball milled for 9 h. (**a**) SEM micrograph; (**b**) mapping of nickel; (**c**) aluminum; and (**d**) oxygen [71].

Arif and Saheb [71] prepared homogenous Ni-Al_2_O_3_ nanocomposite powders with uniform distribution of Al_2_O_3_ nanoparticles though ball milling Ni and Al_2_O_3_ powders for 9 h. They used field emission scanning electron microscopy and X-ray mapping to characterize the ball milled powders and Al_2_O_3_ nanoparticles’ dispersion, respectively. Figure 6 shows X-ray mapping of Ni-10 wt%Al_2_O_3_ nanocomposite powder ball milled for 9 h. SEM micrograph (a), mapping of nickel (b), aluminum (c), and oxygen (d).

**Figure 7:**
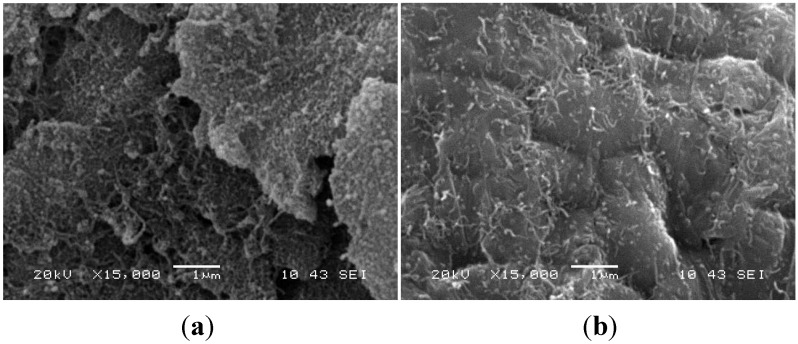
SEM micrographs of Al6061-1 wt% functionalized CNTs powder (**a**) after sonication and (**b**) after sonication and wet ball milling [72].

Saheb *et al.* [72] synthesized CNT reinforced Al6061 and Al2124 alloy based nanocomposites with uniform dispersion of CNTs through sonication and ball milling technique. They reported that the nanocomposite powders prepared through dry milling showed the presence of CNTs in the form of bundles. However, functionalization and sonication of CNTs followed by wet milling led to a uniform dispersion of CNTs. Figure 7 shows SEM micrographs of Al6061-1 wt% functionalized CNTs powder (a) after sonication and (b) after sonication and wet ball milling.

**Figure 8:**
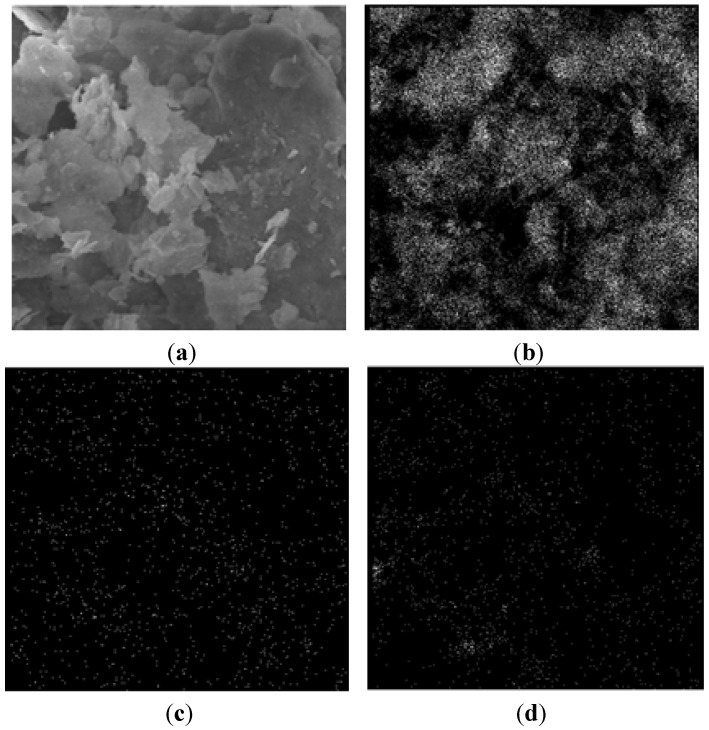
SEM micrographs of Al-1 wt% SiC nanocomposite powder ball milled for (**a**) 24 h; (**b**) mapping of Al; (**c**) Si; and (**d**) O [73].

Saheb *et al.* [73] prepared homogenous Al-1 wt% SiC with uniform distribution of SiC through ball milling for the mixture of powders for 24 h. Figure 8 shows SEM micrographs of Al-1 wt% SiC nanocomposite powder ball milled for 24 h (a), mapping of Al (b), Si (c), and O (d).

**Figure 9:**
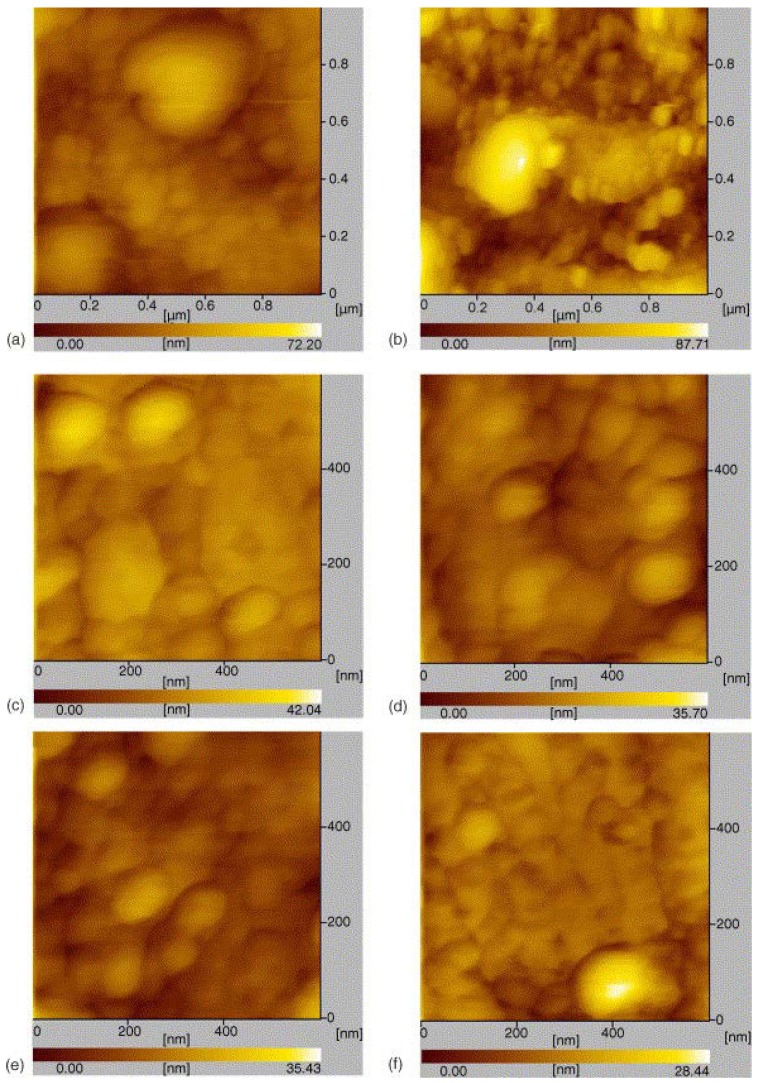
AFM images of characteristic interfacial microstructures at different WC-Co contents (**a**) 20 wt% of WC-10%Co; (**b**) 40 wt% of WC-10%Co, and (**c**–**f**) 30 wt% of WC-10%Co in WC-10%Co particle reinforced Cu matrix nanocomposites [5].

**Figure 10:**
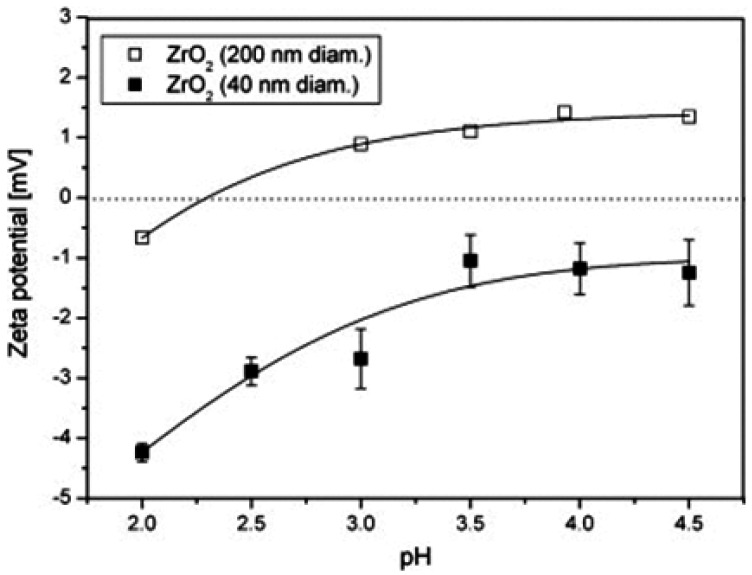
Zeta potential as a function of bath pH, for ZrO_2_ particles of two different average diameters dispersed in the Watts bath [6].

Gu *et al.* [5] prepared WC-10% Co particle reinforced Cu matrix nanocomposites with varying volume fractions of the particles inside the matrix, using Direct Metal Laser Sintering (DMLS) Process. They observed that using 40 wt% of WC-10%Co particles leads to particle agglomeration inside the nanocomposite, while 30 wt% of WC-10%Co particles inside the nanocomposite results in a uniform dispersion of particles inside the Cu matrix. Figure 9 shows the AFM images of characteristic interfacial microstructures of 30 wt% WC-10%Co and 40 wt% of WC-10%Co nanocomposites. As evident in Figure 10, the 40 wt% of WC-10%Co nanocomposite sample shows clear particle agglomeration while uniform particle dispersion can be visualized in the 30 wt% of WC-10%Co nanocomposite sample.

Simunkova *et al.* [6] codeposited nano- and submirco-scaled ZrO_2_ particles of two different sizes (average diameters of 40 nm and 90 nm) into Ni matrix by an electrochemical plating process using a Watts bath. The ZrO_2_ particles were characterized through zeta potential electro-acoustic measurements and particle size distribution. It was observed that, for average particle diameter of 40 nm, a uniform dispersion of ZrO_2_ particles in the Ni matrix was obtained if deposited in the Ni coating when the pH value of the plating bath was equal to 2, while at pH equal to 3.5, an agglomeration of ZrO_2_ particles in the Ni coating was observed. A value of pH equal to 2 was found to correspond to the highest value of the zeta potential measured for particles with 40 nm average diameter, causing high repulsive forces between the particles and consequently a low degree of agglomeration in the plating bath. The low agglomerated particles are further better distributed in the co-deposited Ni-ZrO_2_ layer. However, for particles with average diameter of 200 nm, pH values of 2 and 3.5 did not show a noticeable difference concerning the degree of particle dispersion in the plating bath. However, for a pH value of 3.5, the particles with average diameters of 200 nm were found to be better dispersed in the Ni matrix due to higher surface energy of the particles with average diameter of 40 nm, leading to a greater degree of agglomeration in the plating bath. Figure 10 shows plots of zeta potential as a function of pH, for the two different sizes of ZrO_2_ particles dispersed in the Watts bath. Since the electrochemical deposition of Ni and ZrO_2_ particles was performed simultaneously with the zeta potential measurement of ZrO_2_ particles in the Watts bath, the quality of dispersion of particles in the plating bath, as well as in the Ni matrix is expected to be the same. Furthermore, since this dispersion is exhibited collectively by all the ZrO_2_ particles being deposited in the Ni matrix, it can be safely concluded that zeta potential measurement is a technique, which can be used to analyze the overall quality of nanoreinforcement dispersion in the bulk nanocomposite.

**Figure 11:**
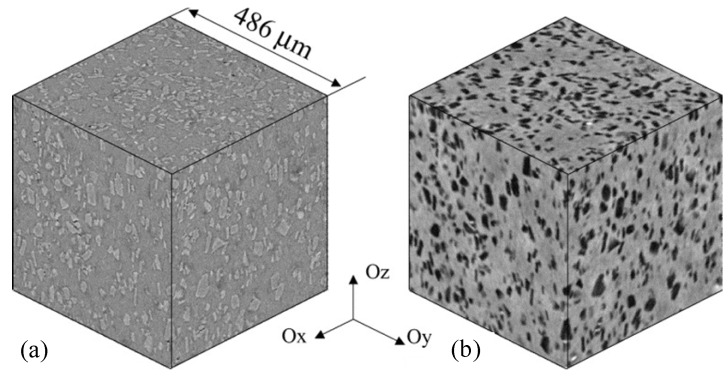
Reconstructed microstructure of 20 vol% Al_2_O_3_ reinforced Al 6061 MMC with two types of X-ray microcomputed tomography. (**a**) Phase-contrast tompography; and (**b**) holotomography [66].

Borbely *et al.* [66] investigated the microstructure of a Al_2_O_3_ particle reinforced Al-6061 matrix composite fabricated via stir casting technique, using two different types of X-ray microcomputed tomography namely high resolution absorption tomography (or holotomography), and phase contrast tomography. The shape of the Al_2_O_3_ particles was approximated by equivalent ellipsoids with the same moment of inertia as the real Al_2_O_3_ particles embedded in the Al-6061 matrix. Figure 11 shows the reconstructed microstructure of the same composite volume, obtained using each of these techniques, where the homogeneous distribution of the ceramic particles in the metal matrix can be easily seen. It is evident from this figure that the image of the composite reconstructed using holotomography depicts a clearer distinction between the particle and the matrix phases, compared to the image reconstructed using phase contrast tomography. Although X-ray microcomputed tomography has not been reported so far with reference to its application towards analysis of nanoreinforcement distribution in a metal matrix nanocomposite, it can be easily deduced from [63,74] that this technique exhibits full potential to reconstruct three-dimensional images of the bulk volume of a nanocomposite, revealing distinct spatial distribution of the embedded nanoreinforcement phase.

The extent of reinforcement dispersion in selected MMNCs is presented in Table 2. In addition, included in the table is information on the fabrication process, the characterization technique used to analyze the nanoreinforcement distribution in the nanocomposite in powder or bulk form, and the quality of nanoreinforcement dispersion reported, as indicated by the respective technique.

**Table 2:** Extent of reinforcement dispersion in selected metal matrix nanocomposites (MMNCs).

Nanocomposite	Fabrication process	Characterization technique	Composite form	Dispersion quality	Reference
SiC/Al	high energy ball milling; plasma activated sintering	SEM and TEM	bulk	well-dispersed	[35 ]
CNT/Cu	molecular level mixing and vacuum sintering	TEM	bulk	well-dispersed	[34 ]
Al_2_O_3_/Fe	high frequency induction heated sintering	SEM and X-ray mapping	bulk	well-dispersed	[70 ]
WC-10% Co/Cu	direct metal laser sintering	AFM	bulk	aggregated for 40 wt%; well-dispersed for 30 wt%	[5 ]
ZrO_2_/Ni	electrochemical plating process	zeta potential measurements	powder	well-dispersed for pH value = 2; aggregated for pH value = 3.5	[6 ]
Al_2_O_3_/Al	stir casting	Holotomography and phase contrast tomography	bulk	well-dispersed	[66 ]
Fly ash/Al	wet blending, cold compaction and sintering	SEM and AFM	bulk	well-dispersed	[75 ]
SiC/Al	pressure-die casting	AFM, SEM and X-ray mapping	bulk	not reported	[76 ]
Ni/Al alloy	blending, cold-pressing and extrusion	SEM and phase contrast tomography	bulk	particles oriented in the extrusion direction	[77]
Si-Zr/Al	blending, hot-pressing and extrusion	phase contrast tomography	bulk	homogeneous	[78]
SiC/Al alloy	rheocasting and extrusion	phase contrast tomography and SEM	bulk	well-dispersed	[79]
SiC/Al and Ta/Al	melt-stirring	SEM and X-ray mapping	bulk	well-dispersed	[80]
Al_2_O_3_/Al	high-energy ball milling	SEM and X-ray mapping	powder	well-dispersed	[13]
Al_2_O_3_/Al alloy	squeeze casting	SEM and TEM	bulk	well-dispersed and intentionally agglomerated	[81]
Al_2_O_3_/Fe-Cr	high-energy ball milling and pulsed current activated sintering	SEM and X-ray mapping	bulk	well-dispersed	[82]
CNT/Cu	molecular level mixing and spark plasma sintering	SEM and TEM	bulk	well-dispersed	[83]
Cu-Ni	reduction of mixed metal oxides	SEM	powder	mix of agglomerates and dispersed particles	[12]
CNT/W-Cu	wet ball-milling and hot-pressing	SEM	bulk	well-dispersed	[84]
W/Cu	wet ball-milling of oxide powder and reduction	SEM and TEM	powder	well-dispersed	[85]
AlN/Al alloy	cryomilling and hot-pressing	TEM	bulk	well-dispersed	[86]
Al_2_O_3_/Cu	wet chemical processing, cold pressing, and pressureless sintering	TEM, STEM, X-ray mapping	bulk	well-dispersed	[14]
CNT/Al	ultrasonication, wet ball-milling, cold compaction and sintering	SEM, TEM and X-ray mapping	bulk	well-dispersed	[87]

Analysis of Table 2 shows that SEM and TEM have been the most frequently used techniques for characterization of nanoreinforcement distribution in metal matrix nanocomposites. However, due to the inherent limitation of microstructural area that can be examined using each of these techniques, subtle conclusions cannot be drawn about the bulk nanoreinforcement distribution in the volume of nanocomposite as a whole. Hence, there exists still an immediate need for more general use of three-dimensional nano-imaging techniques for effective characterization of nanoreinforcement distribution, which can then be used as a tool for a more reliable prediction of bulk mechanical properties of metal matrix nanocomposites.

### 2.3. Ceramic Matrix Nanocomposites

As mentioned above for metal matrix nanocomposites, characterization techniques that can be utilized for analyzing nanoreinforcement distribution in ceramic matrix nanocomposites can either be applied to assess the quality of nanoreinforcement dispersion when the nanocomposite is in powder form or in consolidated form. Characterization techniques for analyzing qualitatively the nanoreinforcement distribution in ceramic matrix nanocomposites, along with the physical form the nanocomposite (powder or consolidated or thin film) to which each of the technique is applicable, are presented in Table 1.

**Figure 12:**
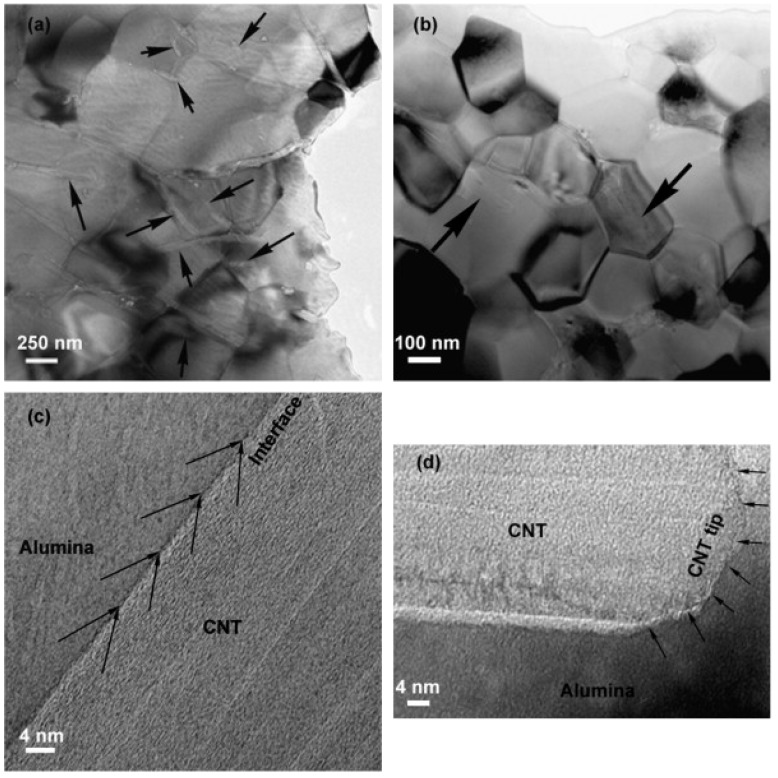
(**a**) and (**b**) FESEM micrographs; (**c**) and (**d**) TEM micrographs of fracture surface of as-consolidated 3.5 vol% CNT reinforced Al_2_O_3_ matrix nanocomposite, black arrows in (**a**) and (**b**) indicate CNTs [88].

Estili *et al.* [88] successfully fabricated 3.5 vol% CNT reinforced Al_2_O_3_ matrix nanocomposites using molecular level mixing process for the fabrication of nanocomposite powders, followed by consolidation using *in situ* Spark Plasma Sintering (SPS) process. Figure 12 shows the SEM and TEM micrographs of the as-consolidated nanocomposites, where a homogeneous dispersion of CNTs can be easily visualized in each micrograph.

**Figure 13:**
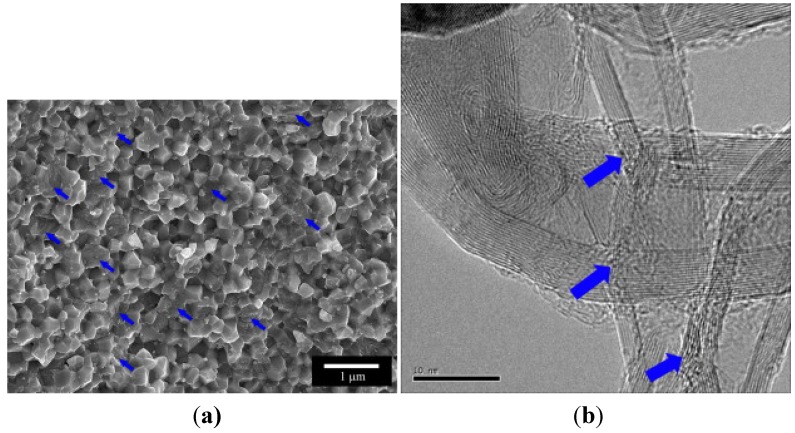
(**a**) SEM micrograph of fracture surface, scale bar: 1 µm; and (**b**) TEM micrograph of sintered sample of CNT reinforced Yttria-stabilized Zirconia (3YZTP) nanocomposite, scale bar: 10 nm. Blue arrows indicate CNTs [89].

Amit *et al.* [89] fabricated CNT reinforced Yttria-stabilized Zirconia (3YZTP) nanocomposites by direct *in situ* growth of CNTs on the Zirconia particles using Chemical Vapor Deposition (CVD), followed by densification via the Spark Plasma Sintering process. The authors reported that CNTs were uniformly distributed in the entire ceramic matrix due to the direct *in situ* growth of CNTs on the powder particles. SEM and TEM micrographs showing homogeneous distribution of CNTs in the ceramic matrix are presented in Figure 13.

**Figure 14:**
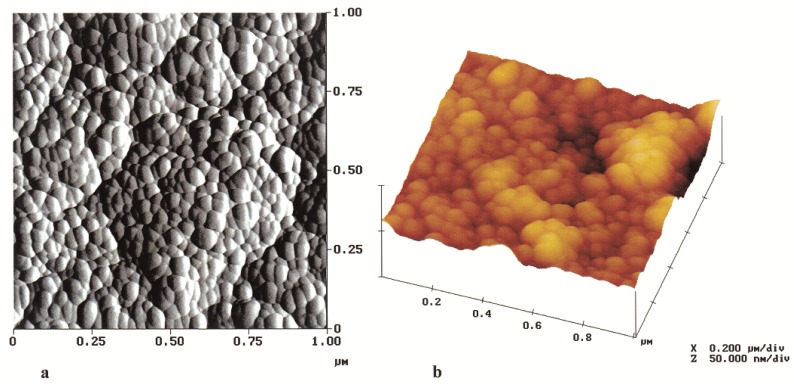
AFM images of Al_2_O_3_ nanocomposite thin film reinforced with NdAlO_3_ nanocrystallites, deposited on a Si target in (**a**) friction (contact mode) and (**b**) 3D (tapping mode) mode [44].

**Figure 15:**
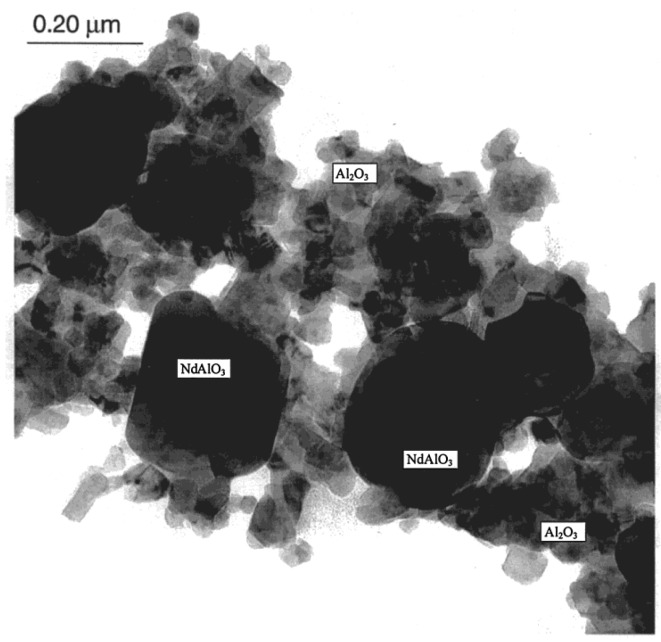
TEM micrograph of the Al_2_O_3_ nanocomposite thin film reinforced with NdAlO_3_ nanocrystallites, deposited on a Si target by CVD and sintered at 1400 °C [44].

Veith *et al.* [44] prepared Al_2_O_3_ nanocomposite thin films reinforced with NdAlO_3_ nanocrystallites using CVD process, starting from a single-source metal-organic precursor containing preformed Nd-O-Al bonds. The thickness of the film was reported to be in the range of 500 nm to 10 µm, deposited on different substrates at a temperature of 500 °C. The AFM images, in friction (contact mode) and 3D (tapping mode) modes of a film deposited on a Si target, are shown in Figure 14. Both types of AFM images shown in Figure 14 reveal a highly uniformly dispersed morphology of NdAlO_3_ nanocrystallites inside the Al_2_O_3_ film, which is also validated by the TEM micrograph of the sintered film shown in Figure 15.

**Figure 16:**
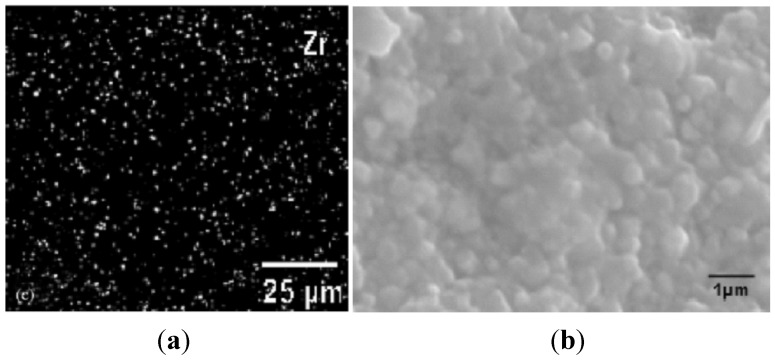
(**a**) X-ray mapping of Zr carried out using EPMA on the wear-scar of 6 wt% ZrO_2_ particle reinforced WC nanocomposite after fretting at 10 N load for 100,000 cycles; and (**b**) SEM of the fracture surface of the nanocomposite densified using SPS at 1300 °C for 5 min [39].

Venkateswaran *et al.* [39] fabricated ZrO_2_ particle reinforced WC nanocomposites containing 6 wt%ZrO_2_ particles using SPS process for consolidation of the nanocomposite powder blend. The tribological behavior of the nanocomposite samples was studied using ball-on-flat-type fretting wear tester. In order to investigate the wear mechanisms, the worn surfaces were examined using SEM and Electron Probe Microanalyzer (EPMA). In order to obtain qualitative and semi-quantitative composition of the tribolayer, X-ray mapping of Zr was performed using EPMA on the worn surfaces, which revealed a homogeneous Zr dispersion as shown in Figure 16, and thus indicated a well-dispersed morphology of ZrO_2_ particles inside the nanocomposite. The SEM micrograph also shown in Figure 16 further validated the X-ray mapping result by showing a homogeneous distribution of ZrO_2_ particles in the fracture surface of the nanocomposite.

Ionescu *et al.* [7] fabricated CNT reinforced SiCN nanocomposites by first dispersing the CNTs in a cross-linked polysilazane using roll-mixing, followed by warm-pressing and pyrolysis in Ar atmosphere. The uniformity of CNT dispersion within the ceramic matrix was studied by performing confocal Raman mapping of 60 × 60 µm^2^ sample surfaces of the CNT/SiCN nanocomposites in the region of 1450–1650 cm^−1^. The authors generated confocal Raman maps of three CNT/SiCN nanocomposites containing 2, 5, and 10 vol% CNTs, pyrolyzed at 1100 °C. The effect of increasing CNT content within the nanocomposite on the dispersion status of CNTs was evaluated from the Raman maps and validated through SEM micrographs of the respective nanocomposite samples. The authors concluded that confocal Raman mapping is a more reliable technique for assessment of a more collective nanoreinforcement distribution in nanocomposites, due to the possibility of a relatively larger surface area of the sample that can be examined, compared to a much more localized cross-sectional area that can be visualized in SEM or TEM.

**Figure 17:**
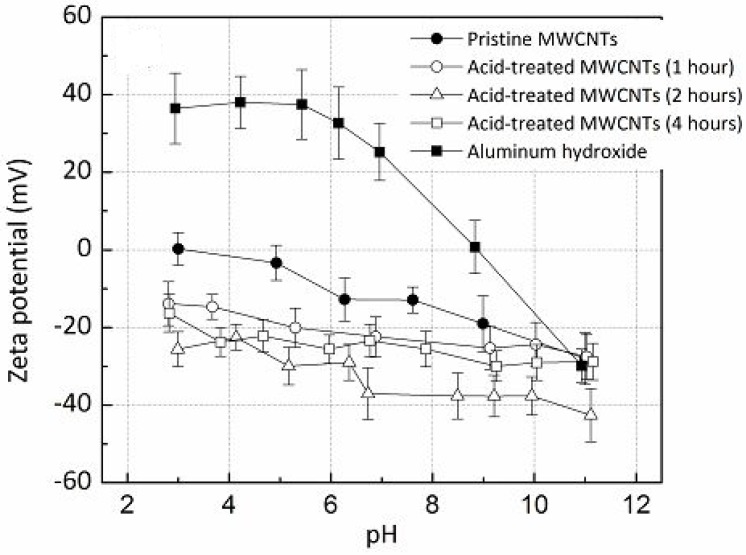
Zeta potential values of MWCNTs and acid-treated MWCNTs at different pH [9].

Yamamoto *et al.* [9] prepared CNT reinforced Alumina matrix nanocomposite via molecular level mixing of functionalized CNTs with Al^3^^+^ ions in ethanol medium, with Al(OH)_3_ used as the Al precursor. Zeta potential measurements of pristine CNTs, acid-treated CNTs, and Al(OH)_3_ were conducted in 1 mM aqueous KCl solution of varying pH using a zeta potential analyzer. Figure 17 shows the zeta potential curves as a function of pH of the KCl solution for pristine CNTs, acid-treated CNTs for different acid-exposure times, and Al(OH)_3_. It is clear from The acid treatment process makes the surface of pristine CNTs more negatively charged at the tested pH range of 2–12, which is mainly attributable to the negatively charged carboxyl and hydroxyl groups introduced on their surfaces by virtue of the functionalization process induced by the acid treatment. This is the reason why the acid-treated CNTs exhibit a much higher degree of dispersion in polar solvents like water and ethanol. The zeta potential of Al(OH)_3_ exhibited positive values over a wide pH range of (pH 3~9), while that of the acid-treated CNTs was measured to be negative in this range. This easily suggests that once the colloidal suspensions of CNTs and Al(OH)_3_ are mixed, particles of Al(OH)_3_ will bind onto the acid-treated CNTs due to the strong electrostatic attractive force between them, resulting in a more homogeneous intermixing of the CNTs and the Al(OH)_3_ particles. Since the same CNT/Al(OH)_3_ suspension mixture is then used for the fabrication of CNT/Al_2_O_3_ nancomposite powders, it can be further deduced that acid-treated CNTs would result in a homogeneous dispersion inside the nanocomposite powder, and hence in the SPS-consolidated nanocomposite. This was indeed validated by the SEM and TEM micrographs of the fracture surfaces of the nanocomposite samples. This finding suggests that zeta potential measurement can be effectively used to assess the quality of nanoreinforcement distribution in the bulk volume of a nanocomposite with a reasonably high degree of precision.

Hazra *et al.* [90] deposited Pt nanoparticles in Al_2_O_3_ matrix on a glass substrate by co-sputtering Pt and Al_2_O_3_ for varying sputtering durations. Grazing Incidence Small Angle X-ray Scattering (GISAXS) of the nanocermet thin films was carried out, using Synchroton source, to predict the shape of the nanoparticles and their distribution inside the Al_2_O_3_ film. The GISAXS results showed that the Pt nanoparticles are ellipsoidal, elongated slightly along the thickness direction, and the average inter-particle separation along the film thickness is greater than that measured in the in-plane direction. It was further verified by specular X-ray reflectivity and diffuse scattering studies of the film, that the Pt particles are randomly distributed, with the degree of randomness increasing along the thickness in the direction of film growth.

The extent of reinforcement dispersion in selected CMNCs is presented in Table 3.

**Table 3:** Extent of reinforcement dispersion in selected CMNCs.

Nanocomposite	Fabrication process	Characterization technique	Composite form	Dispersion quality	Reference
SiO/Graphene	*In situ* chemical synthesis	SEM; TEM	bulk	uniform	[91]
MWCNT/Al_2_O_3_	SPS	TEM; zeta potential	bulk	uniform	[9]
Si_3_N_4_/Graphene	SPS	SEM	bulk	uniform	[92]
CNT/YSZ	*In situ* growth of CNTs on zirconia SPS	FESEM; TEM	bulk	uniform	[89]
Si-C-N/MWCNT	ball milling pyrolysis	SEM; confocal raman mapping	bulk	uniform for 10% CNTs not uniform for 5% CNTs	[7]
CNT/Al_2_O_3_	SPS of individually alumina decorated CNTs	SEM; TEM; Zeta Potential	bulk	uniform for 2.6%–15% CNTs	[11,88]
CNT/Al_2_O_3_	sol-gel process	SEM	bulk	uniform	[93]
Fe/MgO	spray pyrolysis	SEM	powder	uniform	[10]
ZrO_2_/WC	SPS	SEM; X-ray mapping	bulk	uniform	[39]
NdAlO_3_/AL_2_O_3_	CVD	AFM; TEM	bulk	uniform	[44]
Pt/Al_2_O_3_	Co-sputtering	SAXS	thin film	uniform	[90]

As was observed in metal matrix nanocomposites, SEM and TEM have been the most frequently used techniques for characterization of nanoreinforcement distribution in ceramic matrix nanocomposites, as evident from Table 3. Both these techniques only result in producing only a two-dimensional image of a very localized cross-section of the bulk nanocomposite, which cannot be held representative of the collective nanoreinforcement distribution. Moreover, the distribution thus analyzed cannot be used to assess the bulk mechanical properties. Therefore such characterization techniques need to be generally adopted which can be used for efficient a more large-scale analysis of nanoreinforcement distribution, for a more reliable and effective prediction of bulk mechanical properties of ceramic matrix nanocomposites.

## 3. Quantitative Analysis

Despite the consensus that nanoparticle dispersion is one of the key parameters governing the properties of a nanocomposite, the characterization of nanoparticle dispersion reported in literature remains largely qualitative. In this section, some methodologies which may be used for quantitative characterization of nanoparticle dispersion are presented. Many of these techniques have been used to study the dispersion in microcomposites but may be applied to nanocomposites provided microstructure images of sufficient resolution can be obtained (e.g., using TEM).

Methodologies for the quantitative characterization of spatial distribution of a discrete secondary phase can be divided into two broad categories, inter-particle spacing methods and Dirichlet tessellation methods. Inter-particle spacing methods represent the second phase distribution in terms of lengths such as the nearest-neighbor distance or mean-near neighbor distance. The Dirichlet tessellation methods start by dividing the microstructure into polygons in such a way that each polygon only contains a single inclusion. The distribution can then be defined in terms of the parameters of the cells (size, aspect ratio, orientation, *etc.*) or in terms of area ratios as in the local area fraction method. A variety of inter-particle spacing measures may also be derived from a Dirichlet tessellation. In what follows, some of the techniques that may be used to quantify nanoreinforcement distribution are presented.

### 3.1. Local Area Fraction Method

The local area fraction is defined as the ratio of the cross-sectional area of the second-phase particle to the area of the polygon that contains the particle. Mathematically, the distribution can be represented using the probability and the mean value of the local area fraction. The results may also be represented in terms of local area fraction contour plots.

**Figure 18:**
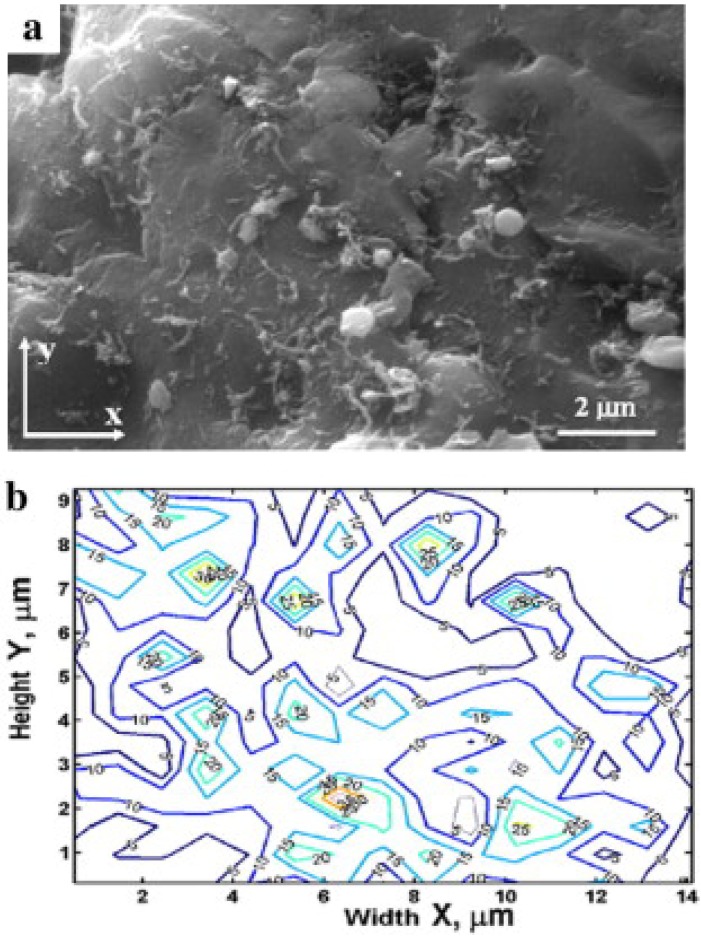
CNT dispersion in aluminum matrix. (**a**) SEM image; and (**b**) local area fraction contour map [97].

Ghosh *et al.* [94] used the mean local area fraction and its standard distribution to quantify the aggregation of the second phase for various numbers of inclusions and the volume fraction of the second phase in a number of computer-generated microstructures. The authors used Voronoi cells resulting from Dirichlet tessellation of a planar heterogeneous microstructure to introduce a unified tool in characterization and response modeling of multiphase materials. Spitzig *et al.* [95] used the cumulative probability distribution and the mean value of the local area fraction to study the dispersion of sulfide and carbide inclusions in steel, and graphite fibers in aluminum composite. The authors compared the actual second-phase particle distributions obtained through automatic image analysis and Dirichlet cell tessellation procedures with computer-generated random particle distributions. They found that the sulfide distributions consisted of clusters superimposed on a random spatial distribution, while the carbide distributions and the graphite-fiber distributions were close to random. Ganguly and Poole [96] used the local area fractions to develop contour maps of the second-phase distributions for a number of computer-generated microstructures. The contour maps were able to distinguish between the different distributions, and capture the position, size, shape and the reinforcement area-fraction in the clusters. Bakshi *et al.* [97] used the contour maps of area fractions to study the dispersion of CNTs in Aluminum composites. Figure 18 shows the SEM image and the local area fraction contour maps for one sample of CNT-Aluminum composite.

### 3.2. Nearest-Neighbor Distance and Near-Neighbor Distance Methods

**Figure 19:**
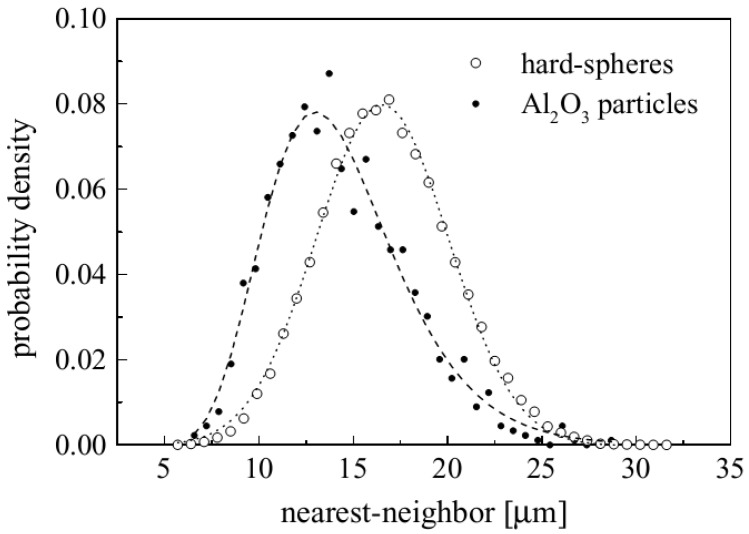
Probability distribution of nearest-neighbor distance [66].

The nearest-neighbor distance is the distance between the centroid of a particle to the centroid of its nearest neighbor. The near-neighbor distance is defined as the average of the distance of the particle’s centroid with the centroids of the particles whose cells share a border with the cell of the concerned particle. Once the nearest neighbor or near neighbor distances have been calculated, the particle distribution can be represented in the form of their mean values along with their probability distribution. Spitzig *et al.* [95] presented another approach to quantify dispersion using nearest-neighbor distance by comparing its mean value and variance with that of microstructures with random dispersion. One problem of the nearest-neighbor method is that if the clustered particles are not properly distinguished during analysis, the nearest neighbor method may lead to incorrect prediction of good dispersion. Despite this problem, the nearest-neighbor method is one of the most widely used methods to quantify dispersion [66,94,95,97,98,99,100,101,102,103]. Figure 19 shows a representative result of the nearest-neighbor method [66].

### 3.3. Mean Intercept Length Method

Another quantitative measure of dispersion derived from the Dirichlet tessellation is the mean intercept lengths in various (e.g., *x* and *y*) directions. The results can be represented in the form of mean intercept lengths *vs.* distance plots or in the form of mean aspect ratios (mean *y*-direction intercept length/mean *x*-direction intercept lengths). Spizig *et al.* [95] used the mean intercept lengths to determine the mean aspect ratios of the Dirichlet cells in steel microstructures and compared the results with aspect ratios of computer-generated microstructures with random dispersion to quantify dispersion. Wray *et al.* [103] used mean intercept length *versus* position plots to measure the degree of dispersion.

### 3.4. Quadrat Method

**Figure 20:**
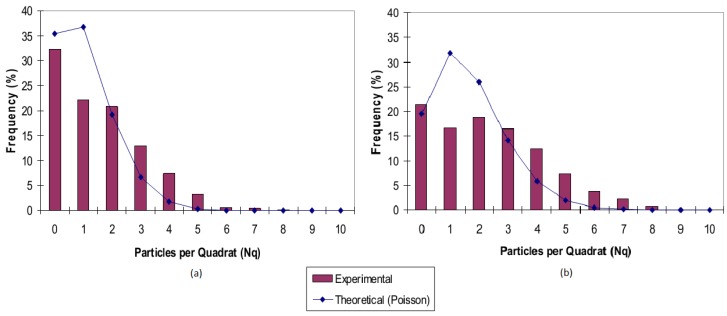
Particle distribution of SiC particles in Zn-Al matrix (**a**) heat treated; (**b**) 35% cold rolled [104].

In the Quadrat method, the microstructure image is divided into a grid of square cells and the number of particles in each cell is counted. The distribution of the number of particles in the cells can then be used to identify random, ordered, or clustered distribution. For example, an ordered distribution would result in a large number of quadrats having the same number of particles, while a clustered distribution would produce some empty quadrats as well as quadrats with a high number of particles. The main difficulty in getting good results using the quadrat method is selection of a correct quadrat size. Karnezis *et al.* [101] used the quadrat method to quantify the dispersion of SiC particles in aluminum alloys. Lucey *et al.* [104] used the quadrat method to study the effects of cold rolling on SiC particle dispersion in Zn-Al alloy. Figure 20 shows a representative result from their work.

### 3.5. Radial Distribution Function

**Figure 21:**
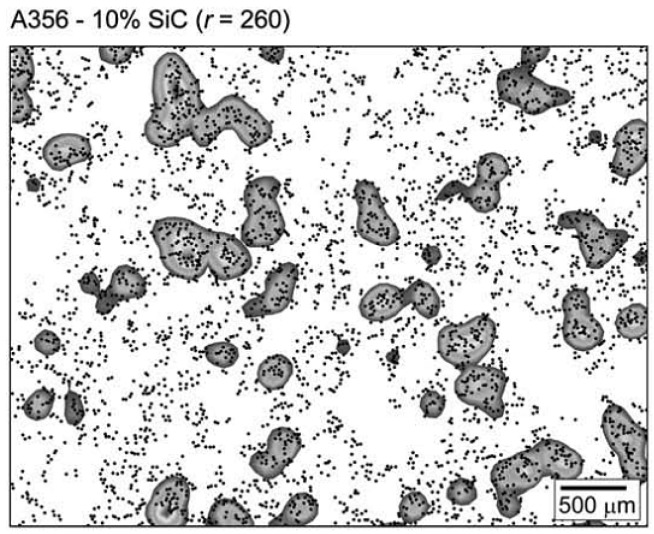
Kernels associated with above-threshold particles for SiC-Al alloy composite [105].

The radial distribution function *H*(*r*) is defined by calculating the ratios of the number of particle centroids that lie within a circle of radius *r*, centered at the centroid of a particle, divided by the particle density of the whole sample. For an ordered arrangement of particles inside the matrix, the function *H*(*r*) will have a value of 1. Moreover, a particle can be regarded as being part of a cluster if its radial distribution function value exceeds a pre-defined threshold value. The threshold value can be determined using computer-generated microstructures. One way to represent the dispersion characteristics within the sample is to plot the mean *H*(*r*) values against the radius *r*. This method was used by Olszówka-Myalska *et al.* [102] for studying the dispersion of alumina particles in aluminum matrix and by Karnezis *et al.* [101] for characterizing the dispersion of SiC particles in an aluminum alloy. Centin and Kalkanli [105] used the radial distribution function to identify clusters in a SiC reinforced Al alloy. The result of their analysis for *r* = 260 µm, for which the maximum fraction of particles were above the threshold, is shown in Figure 21.

### 3.6. Second Order Intensity Function

Second order intensity function estimation not only helps to quantify the distribution of reinforcements inside a composite, but also indicates the average inter-particle distance inside the matrix, as demonstrated by Pyrz [106]. In this method, particles are treated as points, defined by their coordinates within an observation area of the composite. Given *N* distinct particles in a two-dimensional area, a “zone of influence” can be assigned to each particle, consisting of that part of the area which is closer to a selected particle than to any other one. This construction referred to as the Dirichlet tessellation, uniquely defines the immediate neighbors of the particles and divides the area under consideration into continuous polygons. These polygons, using computer-based algorithms, are then made to grow with a uniform rate inside the matrix, until the polygonal edges start to intersect and no further growth is possible. Poisson distribution is then used as a standard to approximate the deviation of the observed distribution of the polygon-enclosed particles, from an “ideal” completely random distribution. This is done by assigning the Poisson distribution as a null hypothesis of complete randomness, and defining distance-based parameters to test the observed particle distribution against the Poisson distribution. Second order intensity function, *K*(*r*), has been observed to be the most effective parameter to test the currently observed particle distribution’s deviation from Poisson distribution [106]. This function is defined as the number of further points that are expected to lie within a distance *r* of an arbitrary point, and divided by the number of points per unit area. Since points which lie outside the current area of observation inside the matrix cannot be observed, pertinent corrections are required in the expression of *K*(*r*), which can finally be defined as [106].

(1) $$K\left(r\right)=\frac{A}{N^{2}}\sum_{k=1}^{N}w_{k}^{-1}I_{k}\left(r\right)$$

where *N* is the number of points in the observation area *A*; *I_k_*(*r*) is the number of points in the circle with center at one of the points and radius *r*; and *w_k_* is the proportion of circumference contained within *A* to the whole circumference with radius *r*.

**Figure 22:**
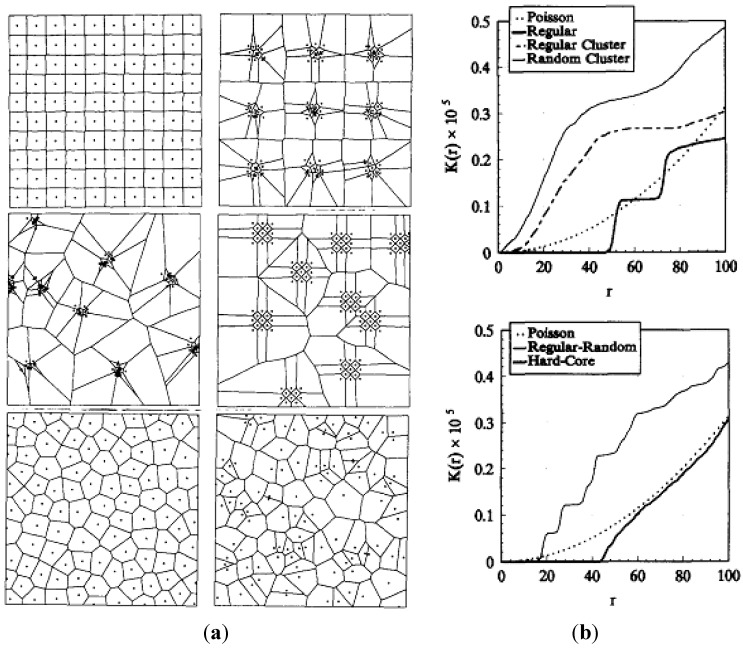
(**a**) Classification of point patterns (from top left to bottom right: regular, regular cluster, random cluster, regular-random, hard-core, Poisson); (**b**) second order intensity function *K*(*r*) for each of the distribution patterns shown in (**a**) [106].

Figure 22a shows some representative examples from a broad classification of the points’ patterns [106]. All plots in this figure are computer simulations of 100 points together with calculated Dirichlet tessellations defined by point distribution. For regular and random cluster patterns shown in Figure 22a, nine cluster centers were distributed accordingly, and offspring points were placed randomly around parent points within the selected matrix area. The regular random distribution was created by placing a regular sub-pattern randomly, while the hard-core model was generated by imposing a minimum permissible distance between any two points which were distributed randomly otherwise [106]. The second-order intensity function *K*(*r*) for all these selected patterns is shown in Figure 22b. The function *K*(*r*) for a completely random Poisson distribution is selected to serve as the standard to distinguish between clustered patterns from those having a certain degree of regularity. As shown in Figure 22b, small- and large-scale distributions (regular and regular-random patterns respectively), show a characteristic stair-like function *K*(*r*) [103]. A horizontal portion of *K*(*r*), for the regular cluster pattern, is used to calculate the average inter-cluster spacing within the matrix. As evident in Figure 22b, the hard-core model lies below the complete random set, suggesting that the corresponding pattern is on the regular side of random.

## 4. Future Directions

The extent of nanoreinforcement dispersion in inorganic nanocomposites is one of the sources of considerable discrepancy between their theoretically predicted and experimentally observed properties. Therefore, future research efforts, in this particular area, could be centered on two important issues, *i.e.*, processing and characterization techniques. In addition to exploring the possibility of introducing innovative processing techniques, there is a strong need to optimize processing parameters of existing methods such as ball milling, ultrasonication, sol-gel, colloidal, and molecular mixing. This will lead to the synthesis of homogenous nanocomposites with uniform distribution of nanoreinforcements and improved properties. The uniform distribution of nano-scale reinforcements should be ascertained with the use of complementary techniques that provides very high resolution such as electron and atomic force microscopy. In addition, further development of three-dimensional nano-imaging techniques, such as X-ray nanotomography, is very much needed to improve their resolution for effective characterization of nanoreinforcement distribution. The possibility to modify and upgrade the well-established quantitative characterization techniques used for conventional composites should be explored to extend their use for nanocomposites.

## 5. Conclusions

A comprehensive review of various characterization techniques used for analyzing nanoreinforcement distribution in inorganic nanocomposites was presented. Methodologies and techniques used to characterize reinforcement dispersion in conventional composites, which may be used for quantitative characterization of nanoreinforcement dispersion in nanocomposites, were also considered. The following conclusions can be drawn from the presented review:

- The SEM and TEM remain the most widely used techniques although both of them cannot be regarded as self-sufficient techniques for a reliable characterization owing to the size limitation of the selected cross-sectional area of the bulk sample which leads to sampling bias in the results.
- Techniques, which provide a broader view of the distribution in the bulk volume, can lead to much more reliable prediction of the properties of nanocomposites. Some of these characterization techniques, which can yield a relatively large-scale distribution analysis in nanocomposites, directly result in a pictorial image of the distribution (for example Confocal Raman Microscopy and Atomic Force Microscopy). Other techniques rely on an indirect analysis for distribution characterization in the bulk volume (like Small-angle X-ray Scattering and Zeta-Potential measurement).
- The X-ray microcomputed tomography is currently only applicable for micro- and macro-composites analysis. However, X-ray nanocomputed tomography may result in a very efficient and reliable three-dimensional pictorial image of the nanoreinforcement distribution within bulk volume of nanocomposite.
- Zeta potential measurement technique offers a two-fold advantage of not only analyzing the distribution of nanoreinforcement in the overall bulk volume of nanocomposite before its consolidation but also yielding a highly reliable measurement of interfacial bond strength between the matrix and the reinforcement phase.
- Qualitative characterization techniques suffer from a common drawback of inability to quantify the distribution of the reinforcement phase within the host matrix—a necessary prerequisite needed for practically feasible evaluation of bulk mechanical properties of nanocomposites.
- Quantitative characterization techniques used for conventional composites involve analyses of microstructure images from which various quantitative measures, either deterministic or probabilistic, are calculated. Deterministic measures include quantities such as mean-intercept lengths in Dirichlet tessellations or local area fractions, while probabilistic measures include probability density functions or frequency distributions in techniques, such as the quadrat method or the nearest neighbor distances.
- There is a potential to modify and upgrade quantitative characterization techniques used for conventional composites to extend their use for nanocomposites.
